# Crystal Methamphetamine Use Predicts Incident STD Infection Among Men Who Have Sex With Men Recruited Online: A Nested Case-Control Study

**DOI:** 10.2196/jmir.6.4.e41

**Published:** 2004-11-29

**Authors:** Sabina Hirshfield, Robert H Remien, Imelda Walavalkar, Mary Ann Chiasson

**Affiliations:** ^2^HIV Center for Clinical and Behavioral StudiesNew York State Psychiatric Institute and Columbia UniversityNew York NYUSA; ^1^Medical and Health Research Association of New York City IncNew York NYUSA

**Keywords:** Internet, sexually transmitted diseases, methamphetamine, HIV

## Abstract

**Background:**

Among men who have sex with men (MSM), the number of newly diagnosed human immunodeficiency virus (HIV) infections has increased by approximately 60% since 1999. Factors that may be contributing to this resurgence include a widely reported increase in bacterial sexually transmitted diseases (STDs) among HIV-positive and HIV-negative MSM, as well as unsafe sexual practices.

**Objective:**

This research was undertaken to learn more about risk behaviors associated with an incident STD among MSM.

**Methods:**

A nested case-control study was conducted, using data from a cross-sectional Internet survey of MSM (N=2643), which investigated risk behaviors during a 6-month period in 2001. Chi-square and logistic regression methods were used to estimate the likelihood of acquiring an incident STD versus no STD.

**Results:**

Eighty-five percent of the respondents were white, 46% were under age 30, and 80% had met sex partners online; 7% were HIV-positive. Men with an incident STD were more likely than men without an STD to report drug use (crystal methamphetamine odds ratio 3.8; 95% confidence interval 2.1-6.7; cocaine OR 2.3; 95% CI 1.2-4.2; ecstasy OR 2.2; 95% CI 1.3-3.8; Viagra OR 2.1; 95% CI 1.2-3.7), alcohol before or during sex (OR 1.9; 95% CI 1.2-2.9), and high-risk sexual behavior (unprotected anal intercourse OR 5.0; 95% CI 2.8-8.9; multiple sex partners OR 5.9; 95% CI 2.5-13.8). In the multivariate analysis, significant independent predictors associated with an incident STD were crystal methamphetamine use (adjusted OR 2.0; 95% CI 1.1-3.8), unprotected anal intercourse (adjusted OR 3.4; 95% CI 1.9-6.3), and 6 or more sex partners during the study period (adjusted OR 3.3; 95% CI 1.4-7.8).

**Conclusion:**

Identifying and treating MSM who have STDs, or who are at increased risk for acquiring STDs, is crucial in preventing the further spread of disease. In addition, there is a need to integrate HIV/STD and substance use prevention and education into Web-based and community-based venues.

## Introduction

Among men who have sex with men (MSM), the number of newly diagnosed human immunodeficiency virus (HIV) infections has increased by approximately 60% since 1999 [1]. Several factors may be contributing to the increase in HIV transmission. One is the widely reported increase in bacterial sexually transmitted diseases (STDs), namely syphilis and gonorrhea, among MSM [2,3]. Not only are STDs a marker for unsafe sexual behavior, but ulcerative and non-ulcerative STDs facilitate the transmission and acquisition of HIV [4,5] and increase HIV viral load and infectivity in persons with HIV [6,7]. Moreover, studies have found a high proportion of HIV-positive MSM with incident STDs [8,9], suggesting continued unsafe sexual practices and exposure of others to HIV. Substance use has also been associated with sexual risk behaviors among MSM [10-12].

Crystal methamphetamine (crystal) use in MSM communities has been problematic in the Western US since the early 1990s [13,14], and has more recently spread to the Midwest [15], as well as the East Coast [16]. Crystal use is associated with “marathon sex” (prolonged sexual activity), receptive and insertive anal sex without a condom, the ability to have sustained arousal for multiple partners, and unsafe sex with HIV-serodiscordant partners or partners of unknown HIV serostatus [15,17,18]. Thus, substance use and its relationship to high-risk sexual behavior among MSM is of particular concern, as drugs may help men to avoid feelings of anxiety associated with same-sex behavior and their own awareness of HIV risk [17,19,20].

This research was undertaken to learn more about risk behaviors associated with an incident STD among MSM. We compared sexual and drug use behaviors between men with a self-reported incident bacterial or viral STD and men without an STD.

## Methods

**Figure 1:**
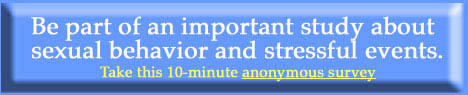
Survey banner ad

We conducted an anonymous, cross-sectional Internet study, inquiring about sexual and drug-using behaviors among MSM between June and December 2001, as part of a larger study of behavior change pre- and post-September 11, 2001. The banner ad (Figure 1) linking to the survey was posted in the online chat rooms of a general interest, gay-oriented website between June 3 and July 24, 2002. Overall, 2284 individuals clicked on the banner but exited the survey without answering any questions; 3697 clicked on the banner and answered the survey. A total of 2949 questionnaires were complete enough for statistical analysis (79% completion rate): 2934 were completed by men (18 of whom were exclusively heterosexual), 10 by women, and 5 by transgendered individuals. Analysis was limited to the 2643 men who reported sex with other men or who self-identified as gay or bisexual, excluding those who never had sex, those who were missing information on STDs, and those who had chronic viral STDs. To assess incident STDs, the questionnaire asked if the respondent had been diagnosed with any STDs during two consecutive 3-month periods between June and December 2001 and provided a checklist of the most common infections. For men reporting viral STDs, we included only viral STDs that were reported in the second 3-month period. Overall, 102 (4%) men reported being diagnosed with an incident bacterial or viral STD during the 6-month study period.

In order to minimize non-valid data, we incorporated reliability checks into the survey for age and certain risk behaviors. To reduce the likelihood of participants' completing multiple surveys, the study banner was rotated through the online chat rooms approximately every 20 minutes. Also, it was not technically possible for participants to bookmark the survey, and there were no monetary incentives to complete the survey.

The general interest, gay-oriented website agreed to host the banner in all of its US adult chat rooms. Individuals had to be registered with the website in order to enter chat rooms. The chat room banner provided the only link to the survey. No personally identifying information was collected. The survey did not use cookies and neither collected user IP addresses nor stored them with submitted data. Study participation was limited to those 18 and older, and all participants clicked on an online consent form before gaining access to the anonymous survey. The Medical and Health Research Association of New York City, Inc. (MHRA) institutional review board approved the study.

The survey included information on demographics (age group, race/ethnicity, education, income and residence), and assessment of risk behaviors, such as type of sexual contact (anal, oral, vaginal; with and without condoms) with main and non-main partners, knowledge of partners' HIV status, type of illicit drug use before or during sex, alcohol use before or during sex, how sex partners were met, and HIV testing. Links to STD prevention/treatment websites and mental health hotlines appeared at the end of the survey. Survey questions were adapted from questionnaires used by the investigators in previous studies.

Data analyses were conducted using SPSS 9.0 for Windows [21]. Bivariate categorical data were evaluated using chi-square and odds ratios. Statistically significant bivariate analyses were simultaneously assessed by multiple logistic regression models. To guard against Type I error, we set the *P*-value to .01 in the bivariate analyses, given that controls outnumbered cases almost 25 to 1.

Respondents were asked how many sex partners they had during two distinct 3-month periods. Respondents could only choose one response from a pull-down menu for each time period. Answer choices were none, 1, 2-5, 6-10, 11-20, 21-50, and 51 or higher. This variable was collapsed for the entire 6-month period; men who reported no partners or one were grouped into the first category. Men who reported 2-5 partners were grouped into the second category, and men who reported 6 partners or more were grouped into the third. For this analysis, “multiple sex partners” refers to 2 or more partners during the study period. Regarding unprotected anal intercourse (UAI), respondents were asked about insertive and receptive sex without a condom. The UAI variable represents men who reported any unprotected receptive and/or insertive anal intercourse. Age was categorized in a pull-down menu: 18-24, 25-29, 30-39, 40-49, 50-59, 60 and older. For ease of analysis, age was collapsed into three categories.

## Results

Overall, the study sample was representative of the host website user population. The host website was able to provide several demographic variables on new registrants from the entire site for the month prior to sample recruitment. Most new registrants were male (87%), and the study sample was identical to the site population in age, and similar in educational attainment and by US region. Although two of the demographic variables were significantly different, the findings may be a reflection of the large samples sizes, which can detect small differences.

**Table 1:** Characteristics of study sample and host Internet website

	**Study**	**Host Website**	P*
	**N (%)**	**N (%)**	
**Age**†	n=2599	n=10 124	
18-24	730 (28)	3090 (29)	
25-34	809 (31)	3091 (29)	
35-44	635 (24)	2451 (23)	
45-54	331 (13)	1172 (11)	
55-64	80 (3)	320 (3)	
65 and older (reference)	14 (<1)	74 (<1)	0.168
			
**Education**	n=2633	n=6394	
High school or less (reference)	335 (13)	939 (15)	
Some college	1093 (41)	2578 (40)	
College degree or more	1205 (46)	2877 (45)	0.050
			
**US Regional Breakdown**	n=2874	n=8846	
Northeast	472 (16)	1658 (19)	
CT, ME, MA, NH, RI, VT, NJ, NY, PA			
Midwest	661 (23)	1998 (22)	
IN, IL, MI, OH, WI, IA, KS, MN, MO, NE, ND, SD			
South	1026 (36)	2910 (33)	
DE, DC, FL, GA, MD, NC, SC, VA, WV, AL, KY, MS, TN, AR, LA, OK, TX			
West (Reference)	715 (25)	2280 (26)	0.006
AZ, CO, ID, NM, MT, UT, NV, WY, AK, CA, HI, OR, WA			

^*^ Chi-square goodness-of-fit statistic used.

^†^ Age brackets were recategorized in order to compare to the host website.

Participants resided in all 50 states, roughly in proportion to the population of each state. Less than 1% resided in Guam, Puerto Rico, and a few locations outside the United States. Approximately half (46%) of the study participants were younger than 30 and had at least a college degree (46%). Most were white (85%). Overall, 6% reported crystal use, 7% reported cocaine, 9% reported ecstasy, 9% reported Viagra, and about half (48%) reported drinking alcohol before or during sex. The aforementioned drugs were commonly used before or during sex (over 85% reported these drugs before or during sex). Most (80%) reported meeting new sexual partners online, and most (80%) engaged in sex with multiple partners. The majority (81%) engaged in sex exclusively with men, and 7% were HIV-positive.

Respondents reported newly diagnosed bacterial or viral STDs (n=102), which included syphilis (n=9), genital herpes (n=4), genital warts/anal warts/HPV (n=16), gonorrhea (n=49), hepatitis B (n=2), chlamydia (n=29), and non-gonococcal urethritis (n=24). Sixteen men reported 2 STDs, and 7 reported 3 or more. Many respondents in the STD group made an effort to notify partners of potential exposure. Approximately 30% notified all partners, 26% told some partners but not all, 17% tried to notify their partners but could not locate them, and less than 5% had the health department notify their sex partners; 21% told none of their partners.

Men with new STDs were more likely to be between 30 and 39 years of age than the controls (see Table 2). The bivariate and multivariate analyses of risk correlates for STDs were structured by drug use and behavioral risk categories as there were no demographic differences between cases and controls. Cases were significantly more likely to report drug use before or during sex (crystal, cocaine, ecstasy, and Viagra), alcohol use before or during sex, and sexual risk behaviors (ie, UAI and multiple sex partners) than the controls (see Table 2). Gamma hydroxy butyrate (GHB), poppers (nitrite inhalants), ketamine, and marijuana use were excluded from the analyses, as their use was not statistically different between groups.

**Table 2:** Comparison of demographic and behavioral characteristics of men with incident STDs and controls

(N=2643)	**STD**	**Controls**	P*
**Demographics**	**N (%)**	**N (%)**	
**Age**	**(n=102)**	**(n=2541)**	
18-29	51 (50)	1167 (46)	.073
30-39	33 (32)	695 (27)	.050
40+ (reference group)	18 (18)	679 (27)	--
**Race/Ethnicity**			
White	81 (82)	2126 (85)	.316
Black	3 (3)	51 (2)	.894
Hispanic	5 (5)	140 (6)	.464
Other/mixed race (reference group)	10 (10)	186 (7)	--
**Education**			
High school or less (reference group)	10 (10)	325 (13)	--
Some college	44 (44)	1049 (41)	.384
College degree or more	47 (46)	1158 (46)	.434
**Income**			
Up to $40 000	58 (64)	1375 (60)	.453
$41 000 or more	33 (36)	924 (40)	
**Met Partners Online**			
Yes	87 (87)	1954 (80)	.080
No	13 (13)	492 (20)	
**HIV Status**			
Positive	11 (11)	180 (7)	.157
Negative or unknown	91 (89)	2361 (93)	

^*^ Age, race, and education used logistic regression to calculate the *P*-value. Income, meeting partners online, and HIV status used chi-square to calculate the *P*-value.

To test for multicollinearity, we ran a linear regression with “any STD” as the dependent variable and the drug and behavioral risk variables from the bivariate analyses as the independent variables. The variance inflation factor (VIF) value for each variable was below 1.5, indicating that multicollinearity was not present. We separated the drug and behavioral risk variables for the multivariate logistic analyses into 3 logistic regression models to assess risk correlates for acquiring an incident STD: model 1 comprised crystal, cocaine, ecstasy, Viagra, and alcohol before or during sex; model 2 comprised UAI and number of sex partners during the study; and model 3 (see Table 3) comprised the significant variables from models 1 and 2.

In model 1, only crystal and alcohol before/during sex were predictive of acquiring an incident STD (crystal, OR 2.7, 95% CI 1.2-6.0, P<.05; alcohol, OR 1.6, 95% CI 1.0-2.6, P<.05). In model 2, UAI and having 6 or more sex partners during the study were predictive of acquiring an incident STD (UAI OR 3.9, 95% CI 2.2-7.1, P<.001; 6 or more partners, OR 4.3, 95% CI 1.8-10.1, P=.001). In the final multivariate model (see Table 3), alcohol before/during sex lost significance and UAI, crystal use before or during sex, and having 6 or more sex partners were the strongest predictors of acquiring an incident STD.

In order to assess the potential for HIV transmission, we compared the HIV status of the participants to that of their partners. Among HIV-positive men with multiple sex partners who reported UAI (n=109), 47% reported UAI with HIV-negative/unknown partners only, 43% reported UAI with positive and negative/unknown partners, and 10% reported UAI with positive partners only. Seven of the 8 respondents with an STD in this subgroup reported sex with serodiscordant partners.

**Table 3:** Bivariate and multivariate analyses: factors associated with incident STDs

	**Incident STD**#		**Drug Use and Behavioral Risk**		**Crystal and Behavioral Risk**	
	Yes	No	Bivariate Odds Ratio (95% CI)	*P*	Multivariate Adjusted Odds Ratio* (95% CI)	*P*
	**N (%)**	**N (%)**				
**Drug Use Before/During Sex**§	n=94	n=2411				
Crystal methamphetamine	16 (17)	124 (5)	3.8 (2.1-6.7)	<.001	2.0 (1.1-3.8)	.024
Cocaine	13 (14)	159 (7)	2.3 (1.2-4.2)	.007		
Ecstasy	17 (18)	219 (9)	2.2 (1.3-3.8)	.003		
Viagra	16 (17)	211 (9)	2.1 (1.2-3.7)	.006		
						
**Alcohol Use**	n=98	n=2499				
Alcohol before sex†	62 (63)	1192 (48)	1.9 (1.2-2.9)	.002	1.3 (0.8-2.1)	.207
						
**Behavioral Risk**	n=102	n=2538				
Unprotected anal intercourse‡	88 (86)	1409 (55)	5.0 (2.8-8.9)	<.001	3.4 (1.9-6.3)	<.001
						
**Sex partners**	n=100	n=2494				
0-1 (reference)	6 (6)	503 (20)	--			
2-5	31 (31)	1103 (44)	2.3 (0.9-5.7)	.056	1.6 (0.6-3.9)	.294
6-100+	63 (63)	888 (36)	5.9 (2.5-13.8)	<.001	3.3 (1.4-7.8)	.007

^*^ Adjusted odds ratio = the odds ratio estimated after adjusting for all other variables included in the parsimonious model.

^#^ Note: In model 1 (data not shown), crystal use and alcohol before/during sex were associated with incident STDs. In model 2 (data not shown), UAI and having 6 or more sex partners were associated with incident STDs.

^†^ Sometimes/most of the time

^‡^ Receptive and/or insertive UAI

^§^ Drug use variables are not mutually exclusive

## Discussion

In this case-control study of men recruited through the Internet, strong associations were found between unprotected anal intercourse, crystal use, and multiple sex partners and an incident STD. In the overall sample, 4% reported a diagnosis of an incident bacterial or viral STD during the 6-month study period. The great majority of HIV-positive men with multiple sex partners reported unprotected sex with HIV-negative or status unknown partners, which signifies the continued risk of spreading HIV and other STDs to non-infected individuals [8]. Other studies of HIV-positive men report a range of serodiscordant or potentially discordant sex, from 21% to 49% [18,22,23]. An average of 80% of our sample met sex partners online, and study findings indicate risk comparable to other Web-based studies on recent sexual risk behavior trends among MSM [24-26].

Men who begin having sex with men while on drugs may develop a pattern of using drugs during sexual experiences [27], and certain drugs such as nitrite inhalants (poppers) and crystal may be used specifically to enhance sexual experiences [27]. Impaired judgment due to drug use may lead to unprotected sex, increasing the risk of HIV/STD transmission [13]. It has been hypothesized that substance use may help men avoid feelings of anxiety associated with same-sex behavior and concerns about HIV risk [20]. Reback's report [17] found that MSM used crystal to cope with negative internal messages about gay sexuality, and HIV-positive MSM reported using it to cope with the fear of transmitting HIV. Reback's report also found that most HIV-positive participants reported that they did not disclose their HIV status to casual sex partners as it was their partner's responsibility to use protection or to set behavioral limits.

Certain limitations of this study deserve mention. Our survey was posted on only one gay-oriented website. We do not know whether survey respondents would differ if the survey had been posted on multiple sites or on sites that specifically facilitate meeting sex partners. Minority MSM were underrepresented in the sample; our data suggest that white, non-Hispanic MSM were unintentionally oversampled, as those who have computer skills and access to participate in online sex surveys tend to be younger, wealthier, educated white males [28-30]. STD was self-report only and we did not ask for the site of infection. There may have been underreporting in this sample, as certain STDs, like chlamydia and gonorrhea, are often asymptomatic and go undetected and unreported [31]. This may also be true for certain viral STDs such as genital herpes [32]. Finally, it is not possible to determine whether respondents who participated in this Internet-based survey are representative of MSM who use the Internet, of MSM in general, or of MSM with HIV, since the MSM population has never been enumerated. Despite these limitations, Internet research is an efficient and inexpensive way to reach large samples of high-risk groups.

Identifying and treating MSM who have STDs, or who are at increased risk of acquiring STDs, is crucial in preventing the further spread of disease. The Internet is a necessary and appropriate medium to reach sex-seeking populations for prevention and intervention efforts [33], especially when factoring in increasing numbers of people living with HIV who are resuming sexual activity as a result of improved treatment regimens [34]. Just as bathhouses and shooting galleries have been used to deliver STD prevention messages, Internet-based interventions should be considered for those seeking sex online [35]. Results of preliminary research on Internet HIV prevention for MSM are promising, suggesting that the Internet may be a reliable resource for studying and targeting risk behaviors in MSM [36].

Studies conducted over the past 20 years have found associations between substance abuse treatment and a reduction in HIV risk behaviors [37]. Primary and secondary substance abuse treatment among MSM has been successful, as treatment can affect decisions about sexual behavior uninfluenced by drugs and alcohol [38]. However, treatment remains challenging, and it has been suggested that men need to abstain from drug use and learn skills to meet and initiate sex with men while sober [12]. The rise in crystal use among the MSM population may require a special focus on current substance abuse treatment approaches, such as addressing drugs in exchange for sex, and disclosure of HIV status [17]. In addition, there is a need to integrate HIV/STD and substance use prevention and education into Web-based and community-based venues. Study findings raise questions concerning the spread of disease and the multiple high-risk behaviors, specifically, how drug use is situated within the trajectory to unprotected sex, multiple partners, and ultimately HIV/STD transmission. Additional data are needed to better understand specific pathways between sexual and drug using practices and HIV/STD transmission among MSM.

## Abbreviations

HIV: Human immunodeficiency virus
MSM: Men who have sex with men
STD: Sexually transmitted disease
UAI: Unprotected anal intercourse
